# Combining Abilities and Heterotic Patterns among Early Maturing Maize Inbred Lines under Optimal and *Striga*-Infested Environments

**DOI:** 10.3390/genes13122289

**Published:** 2022-12-05

**Authors:** Gloria Boakyewaa Adu, Baffour Badu-Apraku, Richard Akromah, Frederick Justice Awuku

**Affiliations:** 1 Council for Scientific and Industrial Research (CSIR)-Savanna Agricultural Research Institute (SARI), Tamale, Ghana; 2 International Institute of Tropical Agriculture (UK) Limited, Carolyn House, 26 Dingwall Road, Croydon CR0 9XP, UK; 3 Department of Crop and Soil Sciences, Faculty of Agriculture, Kwame Nkrumah University of Science and Technology, Private Mail Bag, University Post Office, Kumasi, Ghana

**Keywords:** *Striga*, crop improvement, combining ability, heterotic groupings, molecular markers

## Abstract

Information on the general combining ability of inbred lines and the specific combining ability of hybrid combinations is crucial for successful hybrid development. The objectives of this study were to (i) determine the combining ability of thirty selected early maturing maize inbred lines under *Striga*-infested and optimal environments, (ii) classify the inbred lines into heterotic groups using the general combining ability effects of multiple traits (HGCAMT) and the single nucleotide polymorphism genetic distance (SNP- GD) methods, and (iii) assess the effectiveness of the heterotic grouping methods. One hundred and fifty single-cross hybrids were generated from the thirty inbred lines using the North Carolina Design II mating method. The hybrids and six local check varieties were tested across optimal and *Striga*-infested environments in Ghana and Nigeria in 2016 and 2017. The inheritance of grain yield was controlled by the non-additive gene action under both environments and the additive gene action across the two research environments. The non-additive gene action modulated the inheritance of measured traits under *Striga*-infested environments, except for the *Striga* damage syndrome rating at 8 weeks after planting. Maternal effects were observed for most traits in each environment and across environments. The inbred lines TZEI 127 and TZEI 40 exhibited significant and positive GCA male and female effects for grain yield under each environment and across the two research environments, indicating the presence of favorable alleles for yield improvements. The SNP-GD heterotic grouping method was identified as the most adequate in grouping the thirty inbred lines.

## 1. Introduction

Maize (*Zea mays* L.) is a key staple crop in West and Central Africa (WCA). The importance of maize in the human diet is indispensable, as it provides about 19% of the calorie availability on average and contributes significantly to the nutrition and livelihood of many small-scale farmers in WCA [1]. Notwithstanding the tremendous significance of maize, its production is austerely hampered by abiotic and biotic stresses, including *Striga*, drought, and low soil nitrogen. *Striga hermonthica* is one of the key biological factors that cause severe damage and yield losses to cereal crops such as maize, millet, and sorghum in WCA. Flowering *Striga* plants produce millions of tiny seeds per plant and are easily spread by man, animals, wind, and erosion [2,3]. The seeds in the dry state can persist and stay alive for years [4]. Seed germination may only occur when a signal is received from a potential host plant coupled with favorable environmental conditions such as adequate soil moisture and temperature [5,6]. *Striga* parasitism can keep a maize plant from growing well, causing the leaves to fold and wilt and the grain yield to reduce [7]. Various studies have shown that *Striga* damage to maize on farmers’ fields is cultivar-dependent and could result in significant yield losses ranging from 20 to 100% yearly [4,5,6]. Therefore, breeding for maize cultivars with resistance to *Striga* and high grain yield potential has become the major focus for most plant breeding programs in sub-Saharan Africa (SSA). This can be achieved by exploiting the natural genetic variation available in the maize gene pool to develop high-yielding maize varieties that possess the genes for *Striga* resistance or tolerance.

The availability of relevant information on the gene action nature, mode of inheritance, and combining ability of inbred lines is crucial to the success of a hybrid development program. This information provides the plant breeder with the opportunity to determine the genetic potential of the available germplasm or breeding lines, identify superior parents for hybrid development, and identify the most appropriate selection strategies to be adopted in breeding programs [8,9]. Several workers [6,10,11,12] have utilized combining ability analyses to elucidate the nature and mode of inheritance of *Striga* resistance in maize. However, the results have been inconsistent. For example, Abu et al. [13] and Oyekale et al. [14] reported that the inheritance of grain yield and number of emerged *Striga* plants is largely under additive gene action, whereas Annor et al. [15] established the superiority of nonadditive gene action in the inheritance of the same traits. The variations in the reports indicate the dependence of the expression of genes for *Striga* resistance on the germplasm, making gene action determination for new cultivars vital. Heterotic grouping is important for the efficient and effective utilization of maize-inbred lines in a hybrid breeding program. Therefore, for the success of a hybrid development program, there is a need for information on the heterotic groups of newly developed inbred lines [16]. Plant breeders can use this information as a guide in selecting parents for hybrid development and the prediction of hybrid performance. This improves the chances of maximizing the heterosis and improving the breeding efficiency. 

Suwarno et al. [17] reported that although the clustering of inbred lines based on high single-nucleotide polymorphism (SNP)-based genetic distances could contribute to higher heterosis, a high marker-based genetic distance does not guarantee maximum heterosis. Hence, the determination of the influence of SNP-based genetic distances on the hybrid performance of inbred lines used in the present study will inform the selection of parents for hybrid production. Additionally, understanding the nature of the genotype × environment interactions (GEIs) of newly developed hybrids is crucial as far as the specific or broad adaptation of the hybrids is concerned [18]. Therefore, several studies have used combining ability estimates to assign germplasms into heterotic groups [15,19,20,21,22,23,24]. The commonly used methods include (i) the use of the specific combining ability (SCA) effects of the grain yield only, (ii) the combination of both specific and general combining ability effects (HSGCA), and (iii) integrating multiple traits with significant general combining ability effects (HGCAMT). However, heterotic groupings relying on combining abilities are largely influenced by the environment, leading to inconsistencies in the grouping of breeding lines [15]. To deal with these inconsistencies, the use of molecular-marker-based genetic distances (GD) has become a method of choice and is utilized extensively in the classification of maize germplasm into distinct groups [25,26,27]. However, the heterotic grouping of maize germplasms based on marker-based genetic distances has resulted in contradictory results. This is primarily attributed to the differences in the effectiveness of the platforms used for genotyping. The types of markers and germplasms used could also influence the accuracy of the heterotic groups generated [28,29,30]. A study conducted by Suwarno et al. [17] reported that the SNP-GD approach to the heterotic grouping of maize inbred lines was slightly better than the specific combining ability (SCA)-based approach. Among the HSGCA, SCA, and simple sequence repeats genetic distance (SSR-GD) classification methods, Akinwale et al. [23] established that the HSGCA method was the best approach for effectively placing inbred lines into distinct heterotic groups. Similarly, Badu-Apraku et al. [31] found the HSGCA method to be superior in assigning 17 maize inbred lines into heterotic groups compared to the HGCAMT, SNP-GD, and SCA methods under multiple stress environments. In a related study, Badu-Apraku et al. [32] identified the SNP-GD classification procedure as the most efficient method as compared to other methods (HGCAMT, HSGCA, and SCA) in grouping 14 quality protein maize inbred lines under contrasting environments. 

The aims of this study were to (i) determine the combining ability of the grain yield and agronomic traits of selected early maturing inbred lines under *Striga*-infested and optimal environments, (ii) classify the set of inbred lines into heterotic groups using the HGCAMT and SNP-GD methods, and (iii) compare the effectiveness of the HGCAMT and SNP-GD heterotic grouping methods.

## 2. Materials and Methods

### 2.1. Genetic Materials

Thirty maize inbred lines (15 yellow and 15 white-grained) were used in this study. The inbred lines were bred by the International Institute of Tropical Agriculture (IITA) (Table 1). In a preliminary study, the maize inbred lines were screened and selected according to their performance and varying reactions to *Striga*. The 30 inbred lines were used to develop one hundred and fifty (150) single-cross hybrids using the North Carolina Design II (NC II) mating scheme by Comstock and Robinson [33]. The thirty inbred lines were assembled into six sets made up of five parents (inbred line) each. The crosses were performed in a 6 (sets) × 5 (groups) two-way factorial fashion such that a set of parents used as females were mated to another set of parents used as males. The single-cross hybrids were generated in 2015 at the Council for Scientific and Industrial Research—Savanna Agricultural Research Institute (CSIR-SARI), Nyankpala, Ghana.

### 2.2. Field Experiment

Two separate sets of experiments were carried out for two consecutive years in Ghana and Nigeria. In the first experiment, the one hundred and fifty hybrids and six local check varieties (commercial hybrids) were tested at Nyankpala (9°24′ N, 00°59′ W) and Manga (11°01′02.39 N, 00°15′51.67 W) and Mokwa in Nigeria (9°18′ N, 5°4′ E) in Ghana in 2016 and 2017 under artificial Striga infestation conditions. The first experiment was set up using a 12 × 13 lattice design with two replications. The experimental unit comprised a single-row plot measuring 5 m in length. Spacings of 0.75 m and 0.40 m were used for the inter- and intra-row distances, respectively. Each hill was planted with three seeds and thinned to two at 2 weeks after planting (WAP). Each plot was artificially infested with seeds of *S. hermonthica* following the procedure described by Kim [11]. The fertilizer was applied at a rate of 30 kg P/ha, 30 kg N/ha, and 30 kg K/ha at 21 days after planting (DAP). The application was delayed to boost the germination and attachment of *Striga* in the *Striga*-infested plots [11]. Hand weeding was done to control all weeds but not *Striga.*

The second experiment involved the evaluation of the 150 hybrids and six local checks under optimal environments (high-N (90 kg N/ha) and *Striga*-free environments) at Nyankpala and Kwadaso (6°43′ N, 10° 36′ W) in Ghana and Mokwa during the growing seasons of 2016 and 2017. The experimental design and plot size used were the same as those used in the first experiment. The basal fertilizer application was performed at the rate of 60 kg N/ha, 60 kg P/ha, and 60 kg K/ha at 2 WAP. An extra 30 kg N/ha was applied as a top dressing at 4 WAP. To control the weeds, a combination of herbicide application and manual weeding was employed. A pre-emergence herbicide (atrazine) was sprayed after planting to control the weeds at 1.25 kg ai/ha. Subsequently, a post-emergence herbicide (gramoxone) was used, when necessary, at a rate of 1 L per acre.

### 2.3. Data Collection

The phenotypic data for factors such as the days to 50% anthesis, days to 50% silking, ear height, plant height, stalk lodging, root lodging, ear aspect, plant aspect, husk cover, ears per plant, and anthesis–silking interval (ASI) values were collected in all two experiments. In addition, *Striga* emergence counts and host plant damage syndrome ratings were measured per plot in the *Striga*-infested experiments at 8 and 10 WAP. The field weight of ears harvested per plot was measured in kilograms, and the moisture content of the ears harvested per plot was recorded for all experiments. The grain yield per plot in kilograms per hectare was calculated using the field weight of ears harvested per plot, with a shelling percentage of 80% and adjusted moisture content of 15%. The procedures used to measure all parameters listed above followed the description given by Badu-Apraku et al. [34].

### 2.4. DNA Extraction and Genotyping Using SNPs

Fresh young leaves from each of the thirty maize inbred lines were harvested separately from three-weeks-old seedlings and stored in a −80 °C freezer. Before the genomic DNA extraction, each sample was dried in a Labconco Freezone 2.5 L System lyophilizer (Marshall Scientific, Kansas, MO, USA) followed by grinding using a SpexTM Sample Prep 2010 Geno/Grinder (Thomas Scientific, Metuchen city, NJ, USA). The total genomic DNA extraction was accomplished using the DArT protocol (www.diversityarrays.com/files/DArT_DNA_isolation.pdf (accessed on 20 September 2017)). The quality of DNA in each sample was determined using the agarose gel technique followed by quantification using a Nanodrop-1000 Spectrophotometer (Nanodrop Technologies, Wilmington, DE, USA). The SNP genotyping of extracted DNA samples was done using the Diversity Arrays Platform [35].

The library construction, sequencing, and SNP calling were performed at the Diversity Arrays Facility (Canberra, Australia). The filtering of SNP markers was performed to eliminate SNPs with missing data > 20%, heterozygosity rates of greater than 20%, and minor allele frequencies lower than 0.05 ((DArT) pipeline (DArT P/L, Canberra, Australia)). A total of 15,047 filtered SNPs were used for the heterotic grouping of inbred lines.

### 2.5. Statistical Analysis

An analysis of variance (ANOVA) of the phenotypic data for all 156 hybrids was performed for the individual research environments using the general linear mixed (GLM) model procedure in the Statistical Analysis System (SAS) [36]. Subsequently, a combined ANOVA across research environments was performed. In the combined ANOVA, each combination of location and year was treated as a test environment. The genotype was considered as a fixed effect while the genotype by environment interaction, replication within environment, and block within replication and environment were considered as random effects using PROC GLM in SAS version 9.2 with a RANDOM statement with the TEST option [36]. The genotype means were adjusted for block effects according to the lattice design [37]. The means were separated using the LSD method and the adjusted means were estimated with their standard errors.

An analysis of variance involving only the 150 test hybrids excluding the local checks was performed separately for all measured traits under each of the two research environments. A combined analysis of variance was subsequently performed by fitting the general linear model with a type III sum of squares for all measured traits using the PROC GLM in SAS with a RANDOM statement with the TEST option [36]. In the North Carolina Design II analysis (NC II), the entry main effect was partitioned into variations due to male-within-sets, female-within-sets, and female-by-male-within-sets interactions. The main effects of male-within-sets and female-within-sets interactions are measures of the general combining ability (GCA), while the female-by-male-within-sets interaction reflects the specific combining ability (SCA) effect [38]. Approximate F tests [39] were constructed based on the expectations of mean squares and used to test the male and female-within-set mean squares [40].

### 2.6. Heterotic Grouping

The SNP-derived genetic distance methodology and the HGCAMT method [8,20,21,22] were utilized to classify the 30 inbred lines into heterotic groups under *Striga* infestation conditions, optimal conditions, and across research environments. The traits measured on different scales with significant genotypic mean squares for GCA effects were initially normalized with a mean of zero and unit variance. The normalized GCA effects were converted to Euclidean distances followed by a cluster analysis using WARD’S hierarchical clustering procedure available in SAS [36]. Ward’s minimum variance approach was used to reduce the total within-cluster variance. A total of 15,047 filtered SNP-derived genetic distance (GD) matrices were used to build a hierarchy of clusters, such that similar inbred lines were grouped to form clusters called heterotic groups following the procedure outlined above. The similarity matrix for SNP markers was based on the Jaccard similarity coefficient test [41] in the DARwin software [42].

The efficiencies of the two different heterotic grouping methods used in this study were compared based on their breeding efficiencies [21] at each and across the two research environments. This was done by ordering the mean grain yield of the 150 hybrids from the largest value to the smallest value from each and across the two research environments. The mean grain yields of the 150 hybrids used in this analysis are presented by Adu et al. [43]. The complete number of hybrids for each classification method was categorized into inter-group and intra-group crosses. Subsequently, the complete set of hybrids within each of the two main groups was separated into three sub-groups: high-yielding hybrids (yield group 1, with a mean grain yield ranking among the top 50 lines), intermediate-yielding hybrids (yield group 2, with a mean grain yield between the 51st and 100th lines), and low-yielding hybrids (yield group 3, with a mean grain yield between the 101st and 150th lines). The breeding efficiency (BE) was calculated as follows:

$$\mathrm{Breeding}\;\mathrm{Efficiency}=\frac{\left[\frac{\mathrm{HY}\;\mathrm{INTERGH}}{\mathrm{TN}\;\mathrm{INERGH}}\times 100\right]+\left[\frac{\mathrm{LY}\;\mathrm{INTRAGH}}{\mathrm{TN}\;\mathrm{INTRAGH}}\times 100\right]}{2}$$

where HY INTERGH is the number of high-yielding inter-heterotic group hybrids, TN INTERGH is the total number of inter-heterotic group hybrids, LY INTRAGH is the number of low-yielding intra-heterotic group hybrids, and TN INTRAGH is the total number of intra-heterotic group hybrids. The most efficient heterotic grouping method was identified based on the procedure described by Badu-Apraku et al. [9].

## 3. Results

### 3.1. Analysis of Variance of Phenotypic Traits under Striga Infestation and Optimal Environments

Under each of the two research environments, the ANOVA of the 156 hybrids revealed significant differences among the genotypes, environments, and GEI mean squares for all traits except for the ASI, which was not significant for the genotype or GEI mean squares under optimal and *Striga*-infested environments (Tables S1 and S2). Similarly, the mean square for the environment was not significant for the number of emerged *Striga* plants at 8 WAP (STRCO1), while the GEI mean square was also not significant for the number of emerged *Striga* plants at 10 WAP (STRCO2). However, across the *Striga*-infested and optimal environments, there were significant differences among genotypes, environments, and GEI mean squares for most measured traits except for the genotypes and GEI for ASI (Table S3).

Table 2 and Table 3 present the results of the ANOVA of the 150 hybrids excluding the local checks. Under the *Striga*-infested environments, the mean squares due to the environments and sets were significant for most measured traits except for STRCO1 for the environment and the ASI and root lodging for sets (Table 2). The mean squares for GCAm (GCA mean squares of inbred lines used as male parents), GCAf (GCA mean squares of inbred lines used as a male parent), and SCA were significant for most of the measured traits except for the ASI and stalk lodging. The SCA × E and GCAf × E interaction mean squares were significant for grain yield and most measured traits, while the GCAm × E interaction mean squares were significant for the days to silking, plant aspect, and *Striga* damage rating at 8 (STRRAT1) and 10 (STRRAT2) WAP. Under optimal environments, the mean squares of the environments and sets were significantly different for the measured traits except for the ASI and stalk lodging for the sets (Table 3). Similarly, the mean squares of the GCAm, GCAf, and SCA, and the mean squares of the interactions between the environment and the GCAm, GCAf, and SCA were significantly different for grain yield and most measured traits except for the ASI (Table 3). Across optimal and *Striga*-infested environments, the differences among environments were significant for the measured traits (Table 3). The mean squares due to the set, GCAm, GCAf, SCA, GCAm × E, GCAf × E, and SCA × E were significant for grain yield and most measured traits except for the ASI (Table 3).

### 3.2. Proportionate Contributions of Combining Ability Effects of the Inbred Lines

The comparative magnitude of the sum of squares due to the GCA over the SCA sum of squares for each trait under the different research conditions is shown in Table 4. For optimal environments, the total GCA (GCA male + GCA female) sum of squares contributions to the total variation among the hybrids ranged from 32.87% to 61.16%, while those of the SCA ranged from 38.84% to 67.13% (Table 4). The contribution of the SCA sum of squares relative to the total variation among the hybrids was much larger in comparison to the GCA sum of squares for almost all traits studied, except for the days to anthesis and silking and ear and plant heights (Table 4). The contribution of the GCAf sum of squares to the total GCA sum of squares was higher than the contribution of the GCAm for most traits but not for the root and stalk lodging. The total contributions of the GCA sum of squares under *Striga*-infested environments to the total difference among the hybrids ranged from 28.14% to 45.44%, while those of the SCA ranged from 35.33% to 64.61% (Table 4). Except for STRRAT1, the contribution of the SCA to the genotypic variation among the hybrids for the other phenotypic traits was superior to the contribution of the GCA. The GCAf sum of squares contributions for the grain yield, ASI, root lodging, husk, cover, and STRRAT 1 and STRRAT 2 were larger than those of the GCAm under *Striga* infestation conditions. Stalk lodging led to comparable GCAf and GCAm results (Table 4). Across the two research environments, the contribution of the GCA to the genotypic variation among the hybrids for the grain yield, days to anthesis, days to silking, and plant and ear heights was greater than the contribution of the SCA (Table 4). Among these five traits, the GCAf sum of squares was higher than the GCAm for the grain yield and ear and plant heights.

### 3.3. Estimates of General Combining Ability Effects of Grain Yield and Striga Adaptive Traits of the Maize Inbred Lines

The GCA effects of the inbred lines for the grain yield and other agronomic traits under *Striga* infestation conditions and across research environments are presented in Supplementary Table S4. The inbred lines TZdEI 40, TZdEI 124, and TZEI 127 showed outstanding GCA effects for grain yield under *Striga*-infested and optimal environments and across research environments. TZEI 127 was the only inbred line that exhibited significantly both positive GCAf and GCAm effects for grain yield under *Striga*-infested environments. TZdEI 40, TZdEI 216, and TZEI 470 exhibited significant and positive GCAm effects for grain yield under *Striga*-infested environments, while TZdEI 124 showed significant and positive GCAf effects for grain yield under *Striga*-infested environments. TZdEI 124 showed significant and negative GCAf effects for STRCO1 and STRC02. The inbred line TZdEI 216 showed significant and negative GCAm effects for STRRAT1, STRRAT2, and STRCO1. Similarly, TZEI 127 and TZEI 470 showed negative and significant GCAm effects for STRRAT2, STRCO1, and STRCO2.

### 3.4. Heterotic Groupings and Relationships among the Different Heterotic Grouping Methods

**Figure 1:**
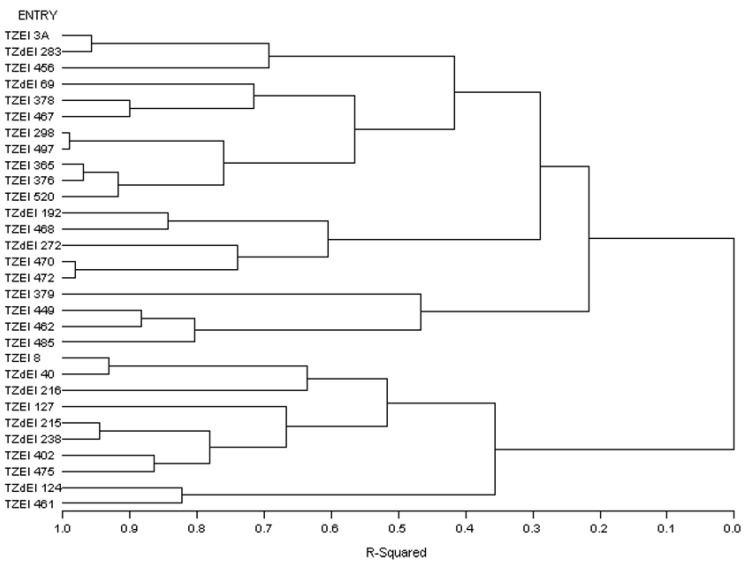
Dendrogram of thirty early maturing maize inbred lines obtained using Ward’s minimum variance cluster analysis based on the HGCAMT effects method across four *Striga*-infested environments, 2016–2017.

**Figure 2:**
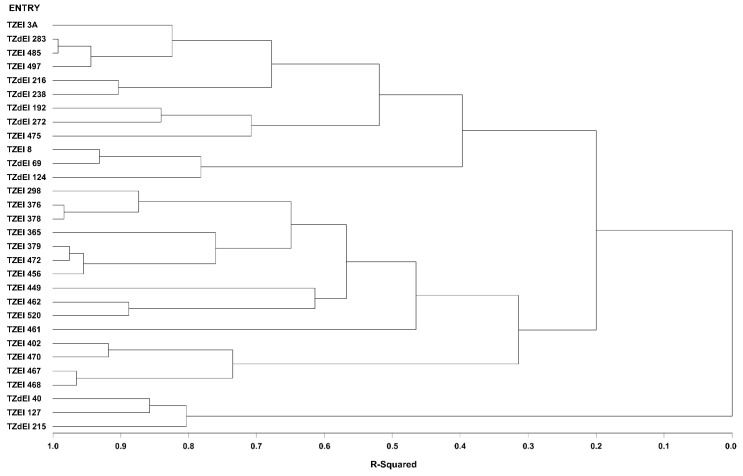
Dendrogram of thirty early maturing maize inbred lines obtained using Ward’s minimum variance cluster analysis based on the HGCAMT method across four optimal environments, 2016–2017.

**Figure 3:**
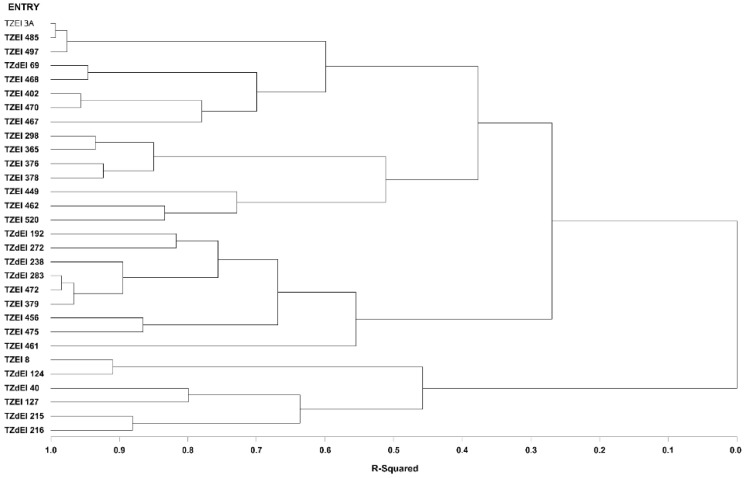
Dendrogram of thirty early maturing maize inbred lines obtained using Ward’s minimum variance cluster analysis based on the HGCAMT method across eight *Striga*-infested and optimal environments, 2016 and 2017.

**Figure 4:**
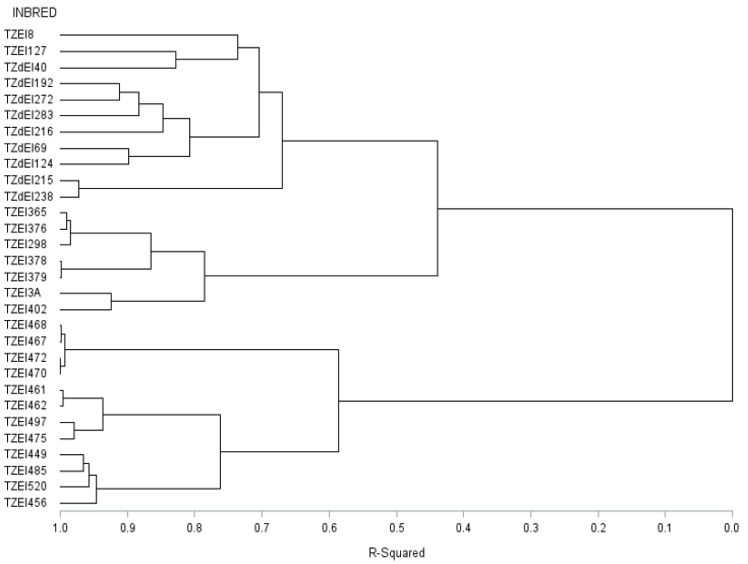
Dendrogram of thirty early maturing maize inbred lines obtained using Ward’s minimum variance clustering method based on 15,047 SNP markers.

The graphical representation and summary of clusters of the 30 inbred lines utilizing the two different heterotic grouping methods are displayed in Figure 1, Figure 2, Figure 3 and Figure 4 and Table 5, respectively. The HGCAMT grouping procedure identified three unique clusters, each under optimal environments and across the two research conditions, and four clusters under the *Striga*-infested environments. In contrast, the SNP-GD method clustered the lines into five groups. Generally, there was very little correspondence between the two different heterotic grouping methods in the clustering of the inbred lines into the same heterotic group (Table 5).

### 3.5. Comparison of Different Methods of Heterotic Grouping under Contrasting Environments

Out of the 150 hybrids studied, the HGCAMT method classified 36, 36, and 37 of them as high-yielding and 30, 18, and 29 as low yielding under optimal, *Striga*-infested, and across the two contrasting environments, respectively (Table 6). The SNP-GD method identified 36, 41, and 43 of the hybrids as high yielding and 22, 25, and 31 as low yielding under optimal, *Striga*-infested, and across environments, respectively (Table 6). The breeding efficiency for the SNP-GD procedure was the topmost under *Striga*-infested conditions (44%), as well as across environments (50.81%), while the breeding efficiency for the HGCAMT methodology was the highest under optimal growing environments (Table 6).

## 4. Discussion

The availability of broad genetic diversity among breeding lines is the most useful indicator of expected genetic gains from selection (Falconer, 1989). The substantial differences observed among the hybrids for yield and other phenotypic traits suggested the presence of sufficient genetic variation for selection gains and the improvement of desirable traits under optimal and *Striga*-infested conditions. This finding is consistent with the reports by Abu et al. [13], Badu-Apraku et al. [44], and Badu-Apraku and Oyekunle [34]. The significant differences observed among the environments indicated that the test environments were diverse and could effectively reveal the variations within the hybrids. The significant GEIs detected for grain yield and other measured traits under optimal and *Striga*-infested environments and across environments implied that the expression of these traits would vary in the different environments [13,34,44]. These results supported the need for rigorous testing of the hybrids in multiple environments and locations over years prior to recommending any of them for commercialization [6].

The significant GCAm, GCAf, and SCA effects for the grain yield and the other measured traits under optimal conditions, *Striga*-infested conditions, and across the two research environments indicated that there were significant variations in performance for the inbred lines as parents in hybrid combinations for those traits. These results indicated that additive and non-additive gene actions were equally crucial in the inheritance of grain yield and other measured traits in each environment and across the research environments. This result corroborates the findings of Badu-Apraku et al. [32]. However, it is inconsistent with the results found by Ifie et al. [45], who reported a non-significant SCA for STRCO under *Striga*-infested environments. The lack of significant GCAm, GCAf, and SCA results for the anthesis–silking interval under the individual conditions and across the two research environments implied that neither additive nor non-additive gene actions modulated the inheritance of the anthesis–silking interval in the test hybrids. This may further suggest that both maternal and paternal effects played a key role in the inheritance of the anthesis–silking interval in the hybrids. The significant GCAf × × E and GCAm × E interactions for most measured traits, including the grain yields under optimal conditions and across environments and for STRRAT1 and STRRAT2 under *Striga*-infested environments, indicated that the GCA variances of the inbred lines were diverse in the different environments. A similar finding was reported by Oyekale et al. [14] for the grain yields and other traits of extra-early biofortified maize inbred lines under optimal conditions and across optimal and *Striga*-infested environments. The non-significant GCAm × E interaction variance observed for the grain yield under *Striga*-infested environments implied that the GCA variances of the inbred lines were stable over the *Striga*-infested environments when the inbred lines were used as male parents. Similarly, the non-significance of GCAm × E and GCAf × E for STRCO1 and STRCO2 indicated that GCA variances of the inbred lines for these traits were consistent over the *Striga*-infested environments when they were used as both male and female parents. The absence of significant SCA × E interaction variances for the anthesis–silking interval, ear height, STRRAT2, STRCO1, and STRCO2 under *Striga*-infested environments suggested that these traits would be stable in specific hybrid combinations under *Striga*-infested conditions. This result is consistent with the results found by Ifie et al. [45], who reported a lack of significant SCA × E interaction variances for grain yield, STRCO1, and STRCO2 under *Striga*-infested environments. This result is also in agreement with the findings of Oyekale et al. [14], who reported the absence of SCA × E interaction variances for the anthesis–silking interval, ear height, STRRAT2, STRCO1, and STRCO2 under *Striga*-infested environments.

Although both additive and non-additive gene actions were important in the inheritance of most measured traits under the target environments, the larger GCA variance obtained over that of the SCA in the present study for the days to silking and anthesis and ear and plant heights under optimal and across environments indicated that the additive gene action largely modulated the inheritance of those traits in the respective environments. Similarly, the results revealed that the inheritance of STRRAT1 and grain yield traits in the test hybrids in *Striga*-infested conditions and across environments, respectively, were largely controlled by the additive gene action. Musila et al. [46] also found the additive gene action to be more important than the non-additive gene action in the inheritance of the days to anthesis for early maturing inbred lines in optimal environments. Furthermore, Konate et al. [47] found the additive gene action to be more important than the non-additive gene action in governing the inheritance of the traits studied in 17 early maturing maize inbred lines across *Striga*-infested and optimal environments. These results implied that early-generation testing would be more effective for selecting for the days to anthesis and silking and ear and plant heights under optimal environments, as well as for the STRRAT1 and grain yield under *Striga* infestation and across environments, respectively. These findings further imply that prediction based largely on GCA variances could aid in the identification and selection of outstanding hybrid combinations [31,32,48]. However, since the SCA variances of the inbred lines were also significant in this study, the GCA variances of the parental lines alone may not be reliable predictors of hybrid performance. In addition, the predominance of the additive gene action suggested that the recurrent selection method is the most appropriate method to use when improving those traits for the test environments [44]. The predominance of the SCA sum of squares over the GCA sum of squares for the grain yield and the other traits under *Striga* infestation indicated that the non-additive gene action is more relevant than the additive gene action in controlling the inheritance of *Striga* resistance or tolerance in the thirty inbred lines used in this study. Kim [11] also found a larger SCA sum of squares compared with the GCA sum of squares for STRCO1 and STRCO2. These results disagree with the findings of Badu-Apraku et al. [31,32], who reported a larger proportion of the GCA sum of squares over the SCA sum of squares for grain yield and most other traits measured under *Striga* infestation conditions, except for days to anthesis and ears per plant. The results are also inconsistent with reports by Oyekale et al. [14], which indicated comparable effects of both additive and non-additive gene actions on the inheritance of grain yield in *Striga*-infested environments. The reports by Gethi and Smith [49], Yallou et al., [50], Badu-Apraku and Oyekunle [34], and Ifie et al. [45] indicated that the additive gene action was more important in the inheritance of STRCO1, and STRCO2 in early maturing maize inbred lines. These reports are also inconsistent with the results obtained in this study. The traits controlled by the non-additive gene action have the highest magnitude of expression of hybrid vigor [51]. Unlike the traits controlled by the additive gene action, those controlled by the non-additive gene action are rarely predicted, as the prediction of such gene combinations has little practical use since they are not transmitted from parents to offspring [52,53]. Therefore, hybrid development could be employed to exploit heterosis to improve the grain yield and desirable traits for *Striga* resistance [54] and superior hybrid performance in *Striga*-infested environments.

The larger sum of squares for the GCAf compared to the GCAm observed for the grain yield and other measured traits under each environment and across environments indicated that maternal effects played a more important role in the inheritance of the grain yield and other measured traits under the respective environments. This finding further suggested a possible role of cytoplasmic gene effects on the measured traits, implying that the choice of the female parent to use in hybridization could influence the selection gains. These findings are consistent with those reported by Derera et al. [55]. These results further corroborated the report by Oyekunle and Badu-Apraku [56], who found maternal genetic effects to be the main factors conditioning grain yield under optimal environmental conditions. Under *Striga* infestation conditions, the inheritance of the days to anthesis and silking, plant and ear aspects, ears per plant, and STRCO1 and STRCO2 traits were controlled by paternal effects. Generally, the disparities in the modes of inheritance of the phenotypic traits observed in the present and previous studies could be attributed to the differences in the germplasms that were utilized.

The combining ability analysis is an important biometric technique for ascertaining the future usefulness and commercial potential of hybrids and their parental lines in hybrid breeding programs [37]. Genetic information on the GCA of breeding lines serves as a guide for the selection of parents and the planning of crosses that would maximize the expression of the grain yield and other desirable traits under target environments. Parental lines with significant and positive GCA effects for measured traits, such that higher values are desirable (e.g., grain yield and ears per plant), imply the potent manifestation of the transfer of desirable allelic variations from the parents to their progenies at high allelic frequencies, while the opposite holds for inbred lines with significant and negative GCA effects. For measured traits such as the *Striga* damage syndrome rating and the number of emerged *Striga* plants, significant and negative GCA effects are rather desirable. The significant and positive GCAm and GCAf effects obtained by TZEI 127 for the grain yields under *Striga*-infested, optimal, and across environments implied that TZEI 127 could contribute favorable alleles to improve grain yields in its progenies under the respective environments when used as either a female or male parent. A similar inference could be made for TZdEI 40, which displayed significant and positive GCAf and GCAm effects for grain yields under optimal growing conditions and across research environments, and for TZdEI 124, which displayed significant and positive GCAf and GCAm effects for grain yields across research environments. Contrarily, TZdEI 40, TZdEI 216, and TZEI 470 would contribute favorable alleles to improve the yield potential of their progenies under *Striga*-infested environments only when used as male parents, while TZdEI 124 would contribute favorable alleles to improve the yield potential of its progenies under *Striga*-infested environments only when used as a female parent. Under *Striga*-infested environments, TZdEI 216, TZEI 470, and TZEI 127 when used as male parents could contribute favorable alleles to produce progenies that will have reduced numbers of emerged *Striga* plants and *Striga* host plant damage. The inbred line TZdEI 124 will produce offspring with a reduced number of emerged *Striga* plants at 8 and 10 WAP when used as a female parent in a hybrid combination with other inbred lines. These results further suggest that TZdEI 124, TZEI 127, TZdEI 216, and TZEI 470 would be desirable in a recurrent selection program and for the development of synthetic populations for improved *Striga* resistance or tolerance and improved grain yields, since the four inbred lines do not only have the potential to reduce *Striga* host damage and *Striga* emergence in their progenies but can also produce progenies with improved grain yields under *Striga*-infested environments [28,43]. Moreover, TZdEI 216 and TZEI 470 could be used to develop outstanding *Striga*-resistant hybrids for commercialization in *Striga*-endemic areas of SSA, while TZdEI 124 and TZEI 127 are ideal parents to develop high-yielding hybrids for non-stress environments, *Striga*-infested environments, and across both contrasting environments. A detailed analysis of the yield potential and stability levels of the single-cross hybrids involving TZdEI 124, TZEI 127, TZdEI 216, and TZEI 470 and the other 26 inbred lines (Table 1) under each and across the eight test environments used in this study was performed by Adu et al. [43]. Furthermore, the observed positive GCA male and female effects for the grain yields of TZEI 127 coupled with its high grain yield per se under each and across the test environments [57] makes TZEI 127 an ideal tester to be used in the determination of the heterotic groups and combining ability of other maize inbred lines.

A heterotic group is a collection of closely related genotypes in such a way that genetically divergent groups are assigned to different groups. Ideally, a cross between inbred lines from different heterotic groups should lead to more vigorous and productive hybrids [58]. Information on the heterotic patterns of the germplasm in a hybrid development program is important, as genetically divergent parents are required to attain the highest expression level of heterosis. The HGCAMT heterotic grouping method clustered the thirty maize inbred lines into three genetically distinct groups, each under optimal growing conditions and across the research conditions. However, four groups were obtained under *Striga*-infested environments. Contrarily, the SNP-GD methods revealed five genetically distinct groups. The differences in the numbers of clusters from the two methods, coupled with the lack of correspondence between the two grouping methods in the assignment of the individual inbred lines into the same heterotic groups, suggested that one of the heterotic grouping methods was more efficient in grouping the inbred lines. The five heterotic groups in the SNP-GD method are of great interest to maize breeders because of the indication that there is high genetic diversity available within the lines to allow significant gains from selection in hybrid development programs. According to Fan et al. [21] and Badu-Apraku et al. [32], a good heterotic grouping method allows for superior hybrids among inter-heterotic group hybridization than intra-heterotic group hybridization programs. Therefore, the highest breeding efficiency obtained for the SNP-GD over the HGCAMT method for the groupings of the thirty inbred lines under *Striga*-infested environments and across environments indicated that the SNP-GD method was the most efficient heterotic grouping method in the present study. The superior performance of the SNP-GD method over the HGCAMT method also suggested that SNP markers could be used to group other inbred lines in other maize programs that are yet to be field tested in hybrid combinations. This result is consistent with the findings of Badu-Apraku et al. [32], indicating the superiority of the SNP-GD method over HGCAMT in the grouping of early maturing inbred lines. This result is also consistent with the reports by Badu-Apraku et al. [24] and Akinwale et al. [23]. It would be more suitable to rely on the grouping based on the SNP-GD method to select parental lines from the inbred lines studied for developing high-yielding hybrids and synthetic varieties. For maximum heterotic effects, crosses should be planned between inbred lines of opposing heterotic groups for the development of productive hybrids with tolerance to *Striga* infestation, and with higher grain yield under optimal and *Striga*-infested environments and across both contrasting environments.

## 5. Conclusions

The inheritance of grain yield under optimal and *Striga*-infested environments were largely controlled by the non-additive gene action, while the additive gene action controlled the inheritance of grain yield across the two contrasting environments. Except for the *Striga* damage syndrome rating at 8 WAP, the inheritance of traits studied under *Striga*-infested environments were mainly modulated by the non-additive gene action. The grain yield and most other traits under and across the research environments were influenced by maternal effects. Maternal effects were also observed for the *Striga* damage syndrome ratings at 8 and 10 WAP under *Striga*-infested environments. The inbred lines TZdEI 124, TZEI 127, TZdEI 216, and TZEI 470 were identified to have good general combining abilities and could be introgressed into maize breeding populations targeted for multiple trait selection and development of commercial hybrids with tolerance to *S. hermonthica*. They could also be used as sources of *Striga* resistance genes that could be introgressed into maize breeding populations. The inbred line TZEI 127 could also be used as a tester to group and determine the combining ability of other maize inbred lines. The SNP-GD heterotic grouping method was superior to the HGCAMT method in grouping the selected set of inbred lines under *Striga*-infested conditions and across environments, while the HGCAMT method was only superior under optimal environments. Therefore, the SNP-GD method was identified as the best heterotic grouping method in this study. However, the practical use of the SNP-GD method would rely greatly on the affordability of the SNP technology and the availability of resources to adequately maintain the relatively large heterotic groups revealed by the SNP markers.

## Figures and Tables

**Table 1:** Description of 30 early maturing maize inbred lines used in North Carolina Design II crosses.

Code	Name	Set	Endosperm Color	Reaction to *Striga* Infestation
1	TZdEI 283	A	White	Tolerant
2	TZdEI 216	A	White	Tolerant
3	TZEI 378	A	White	Tolerant
4	TZEI 379	A	White	Tolerant
5	TZdEI 69	A	White	Susceptible
6	TZdEI 272	B	White	Tolerant
7	TZdEI 215	B	White	Tolerant
8	TZEI 402	B	White	Tolerant
9	TZEI 3A	B	White	Tolerant
10	TZEI 298	B	White	Tolerant
11	TZdEI 238	C	White	Tolerant
12	TZEI 365	C	White	Tolerant
13	TZEI 376	C	White	Tolerant
14	TZdEI 192	C	White	Tolerant
15	TZdEI 124	C	White	Tolerant
16	TZEI 468	D	Yellow	Tolerant
17	TZEI 485	D	Yellow	Tolerant
18	TZEI 461	D	Yellow	Tolerant
19	TZdEI 40	D	Yellow	Tolerant
20	TZEI 520	D	Yellow	Tolerant
21	TZEI 472	E	Yellow	Tolerant
22	TZEI 456	E	Yellow	Tolerant
23	TZEI 462	E	Yellow	Susceptible
24	TZEI 467	E	Yellow	Tolerant
25	TZEI 127	E	Yellow	Tolerant
26	TZEI 475	F	Yellow	Tolerant
27	TZEI 8	F	Yellow	Tolerant
28	TZEI 470	F	Yellow	Tolerant
29	TZEI 449	F	Yellow	Susceptible
30	TZEI 497	F	Yellow	Tolerant

**Table 2:** Mean squares derived from a combined analysis of variance for grain yield and other phenotypic traits of the 150 single-cross hybrids tested under artificial *Striga* infestation conditions in Ghana and Nigeria in 2016 and 2017.

Source of Variation	DF	Grain Yield	Days to Anthesis	Days to Silking	Anthesis–Silking Interval	Plant Height	Ear Height	Root Lodging	Stalk Lodging	Plant Aspect	Ear Aspect	Ear per Plant	*Striga* Damage (8 WAP)	*Striga* Damage (10 WAP)	*Striga* Count (8 WAP)	*Striga* Count (10 WAP)
Environment (E)	3	45,253,715.30 **	2007.65 **	1827.14 **	20.69 **	69452.55 **	4624.55 **	436.32 **	316.83 **	771.40 **	406.07 **	9.21 **	1461.83 **	1778.24 **	0.27	64.41 **
Set	5	2,537,531.50 **	21.03 *	27.40 *	0.33	6836.23 **	760.45 **	0.59	1.78 *	11.68 **	12.98 **	0.36 **	6.79 **	7.23 **	4.64 *	3.37 *
E*Set	15	569,442.60 *	10.26 *	11.90 *	0.23	560.48 *	108.26	1.09 *	0.65	1.95 *	2.03 *	0.06	2.28 **	2.30 *	1.6	1.02
REP (E*Set)	20	549,732.80 *	5.10	4.29	0.21	120.23	46.63	0.59	0.72	0.82	0.82	0.03	0.52	0.98	0.91	0.84
Block (E*REP)	96	772,888.90 **	10.23 **	12.87 **	0.28*	427.12 **	115.41 **	0.92 **	0.99 *	1.57 **	2.11 **	0.05 *	2.23 **	3.41 **	2.02 *	1.15 *
Hybrid	155	693,210.70 **	14.28 **	11.36 **	0.21	879.49 **	165.05 **	0.78 **	0.85 *	2.26 **	1.86 **	0.07 **	1.37 **	1.62 **	2.77 *	2.35 **
GCA-male (Set)	24	741,937.20*	23.49 **	19.48 **	0.13	930.75 **	89.23	0.75 *	0.89	2.40 **	1.96 *	0.08 *	1.83 **	1.40 *	3.37 *	2.99 **
GCA-female (Set)	24	1,002,635.70 **	18.41 **	11.60 *	0.26	962.94 **	212.62 **	1.10 *	0.88	1.91 *	1.87 *	0.06 *	2.02 **	2.69 **	2.23 *	1.84 *
SCA (Set)	96	541,397.90 **	10.79 **	9.23 *	0.22	501.63 **	137.13 **	0.72 **	0.81	1.75 **	1.46 *	0.05 *	0.89*	1.19 *	2.32 *	1.98 **
Hybrid*E	465	461,059.90 *	6.21 *	9.28 *	0.21	264.67 *	64.34	0.67 **	0.74	1.52 **	1.33 **	0.05 *	1.04 **	1.04 *	1.29	0.82
GCA-male (Set)*E	72	376,159.1	5.81	9.07 *	0.21	146.91	43.73	0.55	0.54	1.30 *	1.16	0.04	1.25 **	1.12 *	0.94	0.82
GCA-female (Set)*E	72	608,112.80 *	6.63 *	9.71 *	0.18	260.05	53.88	0.73 *	0.81	2.09 **	1.53 *	0.04	1.47 **	1.23 *	1.45	0.74
SCA (Set)*E	288	442,171.40 *	5.96 *	8.41 *	0.23	266.31 *	67.06	0.67 **	0.79 *	1.32 **	1.18 *	0.05 *	0.83 *	0.88	1.26	0.8
Error	480	330,203.40	4.85	6.92	0.22	200.50	64.60	0.44	0.64	0.76	0.91	0.04	0.62	0.78	1.40	0.84

* Significant at the 0.05 probability level; ** significant at the 0.01 probability level, GCA, general combining ability; SCA, specific combining ability, REP, replication; WAP, weeks after planting.

**Table 3:** Mean squares derived from a combined analysis of variance for grain yield and other phenotypic traits of the 150 single-cross hybrids tested under optimal growing conditions in Ghana and Nigeria in 2016 and 2017 and across eight research environments.

Source of Variation	DF	Grain Yield	Days to Anthesis	Days to Silking	Anthesis–Silking Interval	Plant Height	Ear Height	Root Lodging	Stalk Lodging	Husk Cover	Plant Aspect	Ear Aspect	Ear per Plant
Optimal Environment													
Environment (E)	3	115,159,309.2 **	4466.41 **	2588.8 **	78.71 **	24,827.87 **	4028.33 **	45.29 **	121.96 **	1316.51 **	830.88 **	786.93 **	1.43 **
Set	5	9,415,440.8 **	35.41 **	39.41 **	0.06	26,721.29 **	1615.15 **	2.48 **	0.95	12.19 **	11.75 **	8.01 **	0.17 *
E*Set	15	2,016,314.1 *	8.74 **	8.50 **	0.03	715.28 **	130.08 **	1.69 **	1.23 *	1.2 **	0.87 **	0.49	0.12 *
REP (E*Set)	20	847,607.7	3.07	3.17	0.04	297.9 *	52.55	0.27	0.77	0.48	0.41	0.18	0.13 *
Block (E*REP)	96	1,526,045 **	6.94 **	7.40 **	0.04	417.74 **	141.66 **	0.89 **	0.92 *	0.41 *	0.97 **	0.66 **	0.07
Hybrid	155	3,350,625.4 **	11.68 **	13.43 **	0.04	1914.26 **	292.32 **	1.15 **	1.21 **	1.18 **	1.59 **	1.03 **	0.16 **
GCA-male (Set)	24	3,449,292.5 **	16.70 *	19.33 **	0.04	1665.97 **	271.18 **	1.55	1.76 *	0.99 **	0.93	0.98 *	0.22 **
GCA-female (Set)	24	4,688,082.7 **	17.71 **	21.02 **	0.05	1797.22 **	484.18 **	0.9	1.53 *	0.98	1.73 **	1.25 *	0.23 *
SCA (Set)	96	2,481,527.2 **	6.87 **	7.87 **	0.04	549.83 **	131.25 *	0.97 **	1.06	0.62	1.09 **	0.59 *	0.12
Hybrid*E	465	1,584,497.6 **	5.18 **	6.27 **	0.04	369.54 **	107.02 **	0.91 **	0.93 **	0.65 **	0.75 **	0.47 **	0.12 **
GCA-male (Set)*E	72	1,349,720.6 *	6.80 **	7.86 **	0.03	335.27 **	108.71 **	1.35 **	0.91 *	0.7 **	0.88 **	0.58 **	0.10 *
GCA-female (Set)*E	72	1,703,657.4 **	6.03 **	6.68 **	0.05	390.85 **	138.28 **	0.76	0.84	0.87 **	0.7 **	0.45 **	0.14 **
SCA (Set)*E	288	1,521,403.7 **	4.04 **	5.01 **	0.04	344.79 **	91.04 **	0.79 **	0.93 **	0.53 **	0.67 **	0.43 **	0.12 **
Error	480	975,007	2.78	3.15	0.04	180.54	59.27	0.60	0.68	0.31	0.38	0.30	0.07
Across Research Environments													
Environment (E)	7	205,147,091.00 **	6529.19 **	3624.53 **	46.14 **	75,261.48 **	32,464.35 **	218.13 **	209.89 **	1080.29 **	691.02 **	566.25 **	7.74 **
SET	5	10,372,430.00 **	52.09 **	51.58 **	0.2	29,822.98 **	1847.92 **	1.95 **	2.07 **	6.98 **	22.58 **	20.12 **	0.42 **
E*SET	35	1,333,866.00 **	8.76 **	10.91 **	0.14	1083.02 **	177.6 **	1.32 **	0.9	1.78 **	1.33 **	1.21 **	0.09 **
REP (E*SET)	40	698,670	4.08	3.73	0.12	209.07	49.59	0.43	0.75	0.38	0.62	0.5	0.08 *
Block (E*REP)	192	1,149,467.00 **	8.58 **	10.14 **	0.16 *	422.43 **	128.54 **	0.90 **	0.96 **	0.66 **	1.27 **	1.39 **	0.06
Hybrid	155	2,748,369.00 **	18.80 **	17.29 **	0.13	2271.94 **	314.22 **	1.24 **	1.38 **	0.85 **	2.43 **	2.01 **	0.13 **
GCA-Male (Set)	24	3,024,316.00 **	32.99 **	32.81 **	0.08	2215.06 **	279.84 **	1.80 *	1.67 **	0.63	2.04 **	1.69 *	0.18 **
GCA-Female (Set)	24	4,296,941.00 **	28.29 **	25.5 **	0.15	2312.74 **	571.42 **	1.28 *	1.81 **	0.9	1.95	1.97 **	0.17 *
SCA (Set)	96	1,774,458.00 **	11.16 **	9.64 **	0.13	656.27 **	145.87 **	1.00 *	1.19 **	0.52	1.47 **	1.3 **	0.1
Hybrid*E	1085	1,060,594.00 **	5.90 **	7.73 **	0.13	346.13 **	93.71 **	0.78 **	0.81 **	0.56 **	1.18 **	0.9 **	0.08 **
GCA Male (Set)*E	168	909,546.00 **	6.45 **	8.11 **	0.12	261.2 **	770 *	0.88 **	0.76	0.60 **	1.12 **	0.92 **	0.08 **
GCA-Female (Set)*E	168	1,191,615.00 **	6.54 **	8.02 **	0.12	342.87 **	100.16 **	0.74 **	0.79	0.77 **	1.44 **	1.01 **	0.09 **
SCA (Set)*E	672	1,018,925.00 **	5.21 **	6.84 **	0.14	318.81 **	85.37 **	0.72 **	0.84 **	0.44 **	1.05 **	0.8 **	0.08 **
Error	960	652,605.00	3.81	5.03	0.13	190.52	61.93	0.52	0.66	0.32	0.57	0.60	0.05

* Significant at the 0.05 probability level; ** significant at the 0.01 probability level, GCA, general combining ability; SCA, specific combining ability, REP, replication.

**Table 4:** Proportion (%) of the sum of squares for crosses attributable to the general combining ability (GCA) and specific combining ability (SCA) for grain yield and other phenotypic traits of early maturing inbred lines under optimal environments, *Striga*-infested environments, and across environments in 2016 and 2017.

Trait	Optimal			*Striga*-Infested			Across Environments		
	GCA		SCA	GCA		SCA	GCA		SCA
	Male	Female		Male	Female		Male	Female	
Grain yield	19.10	25.95	54.95	16.57	22.40	48.37	20.97	29.80	49.23
Days to anthesis	26.98	28.61	44.41	25.48	19.96	46.79	31.15	26.71	42.14
Days to silking	26.91	29.27	43.81	26.56	15.82	50.35	33.87	26.32	39.81
Anthesis–silking interval	14.58	18.29	67.13	9.66	18.48	64.61	10.06	20.03	69.91
Plant height	29.42	31.74	38.84	16.39	16.95	35.33	30.97	32.33	36.70
Ear height	21.18	37.82	41.00	8.37	19.95	51.46	19.50	39.83	40.67
Root lodging	24.45	14.14	61.41	14.90	21.74	57.37	25.32	18.07	56.61
Stalk lodging	23.36	20.29	56.34	16.24	16.08	58.74	20.21	22.01	57.78
Husk cover	22.14	22.02	55.84	18.15	21.00	55.15	17.45	24.77	57.79
Plant aspect	13.30	24.75	61.94	16.40	13.04	47.83	20.70	19.74	59.56
Ear aspect	21.39	27.09	51.53	16.31	15.55	48.46	19.08	22.26	58.67
Ears per plant	23.37	24.84	51.79	19.31	14.16	49.28	23.83	21.90	54.28
*Striga* damage syndrome rating at 8 WAP				20.69	22.86	40.16			
*Striga* damage syndrome rating at 10 WAP				13.37	25.66	45.40			
Number of emerged *Striga* plants at 8 WAP				18.82	12.43	51.90			
Number of emerged *Striga* plants at 10 WAP				19.70	12.13	51.98			

**Table 5:** Heterotic groups of the thirty early maturing maize inbred lines based on the HGCAMT and SNP-GD methods under individual and across contrasting environments.

Method	Group 1	Group 2	Group 3	Group 4	Group 5
HGCAMT-*Striga*-infested environment	TZEI 3A, TZdEI 283, TZEI 456, TZdEI 69, TZEI 378, TZEI 467, TZEI 298, TZEI 497, TZEI 365, TZEI 376, TZEI 520	TZdEI 192, TZEI 468, TZdEI 272, TZEI 470, TZEI 472	TZEI 379, TZEI 449, TZEI 462, TZEI 485	TZEI 8, TZdEI 40, TZdEI 216, TZEI 127, TZdEI 215, TZdEI 238, TZEI 402, TZEI 475, TZdEI 124, TZEI 461	
HGCAMT-Optimal environment	TZEI 3A, TZEI 497, TZdEI 283, TZEI 462, TZdEI 216, TZdEI 238, TZEI 485, TZEI 402, TZEI 472, TZEI 402, TZEI 456, TZEI 298, TZEI 461	TZdEI 192, TZdEI 272, TZEI 475, TZEI 449, TZEI 365, TZEI 379, TZEI 520, TZEI 378, TZEI 467, TZEI 468, TZEI 470	TZEI 8, TZdEI 124, TZdEI 69, TZdEI 40, TZEI 127, TZdEI 215		
HGCAMT-Across environment	TZEI 3A, TZEI 462, TZdEI 192, TZEI 475, TZdEI 238, TZEI 402, TZdEI 283, TZEI 472, TZEI 379, TZEI 456, TZEI 468	TZdEI 69, TZdEI 272, TZEI 485, TZEI 497, TZEI 376, TZEI 470, TZEI 467, TZEI 461, TZEI 298, TZEI 378, TZEI 365, TZEI 520, TZEI 449	TZEI 8, TZdEI 124, TZdEI 40, TZEI 127, TZdEI 215, TZdEI 216		
SNP-GD	TZEI 8, TZEI 127, TZdEI 40, TZdEI 192, TZdEI 283, TZdEI 216, TZdEI 69, TZdEI 124, TZdEI 272	TZdEI 215, TZdEI 238	TZEI 365, TZEI 376, TZEI 298, TZEI 378, TZEI 379, TZEI 3A, TZEI 402	TZEI 468, TZEI 467, TZEI 472, TZEI 470	TZEI 461, TZEI 462, TZEI 497, TZEI 475, TZEI 449, TZEI 485, TZEI 520, TZEI 456

**Table 6:** Numbers of inter- and intra-group hybrids categorized by the HGCAMT and SNP genetic distance (GD)-based heterotic grouping methods and the breeding efficiency (BE) of the different methods under optimal, *Striga*-infested, and across environments.

		**Optimal Growing Environments**	
**Yield Group**	**Cross Type**	**HGCAMT**	**SNP-GD**
1	inter-group	36	36
1	intra-group	14	14
2	inter-group	31	30
2	intra-group	19	20
3	inter-group	20	28
3	intra-group	30	22
BE		44.50	38.79
		***Striga*-Infested Environments**	
**Yield Group**	**Cross Type**	**HGCAMT**	**SNP-GD**
1	inter-group	36	41
1	intra-group	14	9
2	inter-group	38	28
2	intra-group	12	22
3	inter-group	32	25
3	intra-group	18	25
BE		37.44	44.13
		**Across Environments**	
**Yield Group**	**Cross Type**	**HGCAMT**	**SNP-GD**
1	inter-group	37	43
1	intra-group	13	7
2	inter-group	31	33
2	intra-group	19	17
3	inter-group	21	19
3	intra-group	29	31
BE		44.56	50.81

## Data Availability

The datasets generated during the present study are available from the lead and corresponding authors on request. A section of the data can be found at: http://data.iita.org/dataset/geneticdiversity-and-population-structure-of-earlymaturiing-tropical-inbred-lines; https://dx.doi.org/10.25502/20181017/0932/AG (accessed on 1 November 2022).

## Supplementary Materials

The following supporting information can be downloaded at: https://www.mdpi.com/article/10.3390/genes13122289/s1: Table S1: Mean squares for grain yield and other phenotypic traits of 156 early maturing single-cross hybrids including local checks evaluated under optimal growing conditions in Ghana and Nigeria in 2016 and 2017. Table S2: Mean squares for grain yield and other phenotypic traits of 156 early maturing single-cross hybrids including local checks evaluated under *Striga* infestation in Ghana and Nigeria in 2016 and 2017. Table S3: Mean squares for grain yield and other phenotypic traits of 156 early maturing single-cross hybrids including local checks evaluated across optimal and *Striga*-infested environments in 2016 and 2017. Table S4: General combining ability effects of 30 inbred lines for grain yields, *Striga* damage ratings, and emerged *Striga* counts evaluated under contrasting environments, 2016–2017.
