# Supplementary material for: Combining Abilities and Heterotic Patterns among Early Maturing Maize Inbred Lines under Optimal and Striga-Infested Environments
Source: Genes (Basel). 2022 Dec 5;13(12):2289. doi: 10.3390/genes13122289 (PMC9778638; doi:10.3390/genes13122289)
Supplement: Supplementary file 1 [file genes-13-02289-s001.zip › Supplementary Table 1.docx]

Supplementary Table 1. Mean squares for grain yield and other phenotypic traits of 156 early maturing single-cross hybrids including local checks evaluated under optimal growing conditions in Ghana and Nigeria in 2016 and 2017.

| Source of variation | DF | Grain yield | Days to anthesis | Days to silking | Anthesis-silking interval | Plant  height | Ear  height | Root lodging | Stalk  lodging | Husk  Cover | Plant  aspect | Ear aspect | Ears per plant |
| --- | --- | --- | --- | --- | --- | --- | --- | --- | --- | --- | --- | --- | --- |
| Environment (E) | 3 | 128930826.6** | 4651.21** | 2672.88** | 83.33** | 25973.74** | 3878.79** | 47.56** | 128.68** | 1397.39** | 868.72** | 836.18** | 1.57** |
| Replication (Rep) | 4 | 11097779.1** | 16.16** | 12.92** | 0.03ns | 978.68** | 493.41** | 1.65* | 3.46** | 1.6** | 11.93** | 2.77** | 0.15ns |
| Block (E x Rep) | 96 | 1537262.1** | 7.49** | 8.4** | 0.05ns | 421.3** | 137.96** | 1.00** | 1.01** | 0.41* | 0.93** | 0.65** | 0.07ns |
| Genotype (G) | 155 | 3350625.4** | 11.68** | 13.43** | 0.04ns | 1914.26** | 292.32** | 1.15** | 1.21** | 1.18** | 1.59** | 1.03** | 0.16** |
| G x E | 465 | 1584497.6** | 5.18** | 6.27** | 0.04ns | 369.54** | 107.02** | 0.91** | 0.93** | 0.65** | 0.75** | 0.47** | 0.12** |
| Error | 524 | 975192 | 2.83 | 3.27 | 0.04 | 192.64 | 61.03 | 0.58 | 0.68 | 0.32 | 0.38 | 0.3 | 0.07 |

^*^, ^**^, Significant at 0.05 and 0.01probability levels, respectively, and ns, non- significant.
