# Supplementary material for: Combining Abilities and Heterotic Patterns among Early Maturing Maize Inbred Lines under Optimal and Striga-Infested Environments
Source: Genes (Basel). 2022 Dec 5;13(12):2289. doi: 10.3390/genes13122289 (PMC9778638; doi:10.3390/genes13122289)
Supplement: Supplementary file 1 [file genes-13-02289-s001.zip › Supplementary Table 2.docx]

Supplementary Table 2. Mean squares for grain yield and other phenotypic traits of 156 early maturing single cross hybrids including local checks evaluated under *Striga* infestation in Ghana and Nigeria in 2016 and 2017.

| Source of variation | DF | Grain yield | Days to anthesis | Days to silking | Anthesis-silking interval | Ear aspect | Ears per plant | STRRAT1 | STRRAT2 | STRCO1 | STRCO2 |
| --- | --- | --- | --- | --- | --- | --- | --- | --- | --- | --- | --- |
| Environment (E) | 3 | 47069185.30^**^ | 2153.65^**^ | 1890.76^**^ | 22.03^**^ | 439.31^**^ | 9.81^**^ | 1567.83^**^ | 1915.68^**^ | 0.20 | 65.98^**^ |
| Replication (Rep) | 4 | 2438688.10^**^ | 16.49^*^ | 19.76^*^ | 0.19 | 7.46^**^ | 0.11^*^ | 3.07^*^ | 23.72^**^ | 20.33^**^ | 21.60^**^ |
| Block (E x Rep) | 96 | 763209.50^**^ | 10.48^**^ | 13.49^**^ | 0.27 | 2.09^**^ | 0.05^*^ | 2.08^**^ | 3.36^**^ | 2.16^*^ | 1.23^*^ |
| Genotype (G) | 155 | 693210.70^**^ | 14.28^**^ | 11.36^**^ | 0.21 | 1.86^**^ | 0.07^**^ | 1.37^**^ | 1.62^**^ | 2.77^*^ | 2.35^**^ |
| G x E | 465 | 461059.90^*^ | 6.21^*^ | 9.28^*^ | 0.21 | 1.33^**^ | 0.05^*^ | 1.04^**^ | 1.04^*^ | 1.29 | 0.82 |
| Error | 524 | 332645.7 | 4.89 | 6.71 | 0.21 | 0.92 | 0.04 | 0.65** | 0.8 | 1.39 | 0.82 |

^*^, ^**^, Significant at 0.05 and 0.01probability levels, respectively, and ns; STRRAT1, *Striga* damage rating at 8 weeks after planting; STRRAT2, *Striga* damage rating at 10 weeks after planting; STRCO1, *Striga* emergence count at 8 weeks after planting; STRCO2, *Striga* emergence count at 10 weeks after planting.
