# Supplementary material for: Combining Abilities and Heterotic Patterns among Early Maturing Maize Inbred Lines under Optimal and Striga-Infested Environments
Source: Genes (Basel). 2022 Dec 5;13(12):2289. doi: 10.3390/genes13122289 (PMC9778638; doi:10.3390/genes13122289)
Supplement: Supplementary file 1 [file genes-13-02289-s001.zip › Supplementary Table 3.docx]

Supplementary Table 3. Mean squares for grain yield and other phenotypic traits of 156 early maturing single-cross hybrids including local checks evaluated across optimal and *Striga*-infested environment in 2016 and 2017.

| Source of variation | DF | Grain yield | Days to anthesis | Days to anthesis | Anthesis- silking interval | Plant height | Ear height | Root lodging | Stalk lodging | Husk  Cover | Plant aspect | Ear aspect | Ears per plant |
| --- | --- | --- | --- | --- | --- | --- | --- | --- | --- | --- | --- | --- | --- |
| Environment (E) | 7 | 224450021.00** | 6865.90** | 3772.06** | 48.81** | 80093.48** | 35008.26** | 232.56** | 224.63** | 1152.96** | 728.91** | 607.65** | 8.24** |
| Replication (Rep) | 8 | 6768234.00** | 16.32** | 16.34** | 0.11ns | 921.24** | 445.34** | 1.08* | 3.24** | 6.62** | 8.55** | 5.12** | 0.13* |
| Block (E x Rep) | 192 | 1150236.00** | 8.99** | 10.95** | 0.16* | 442.4** | 131.77** | 0.97** | 1.00** | 0.7** | 1.26** | 1.37** | 0.06ns |
| Genotype (G) | 155 | 2748369.00** | 18.80** | 17.29** | 0.13ns | 2271.94** | 314.22** | 1.24** | 1.38** | 0.85** | 2.43** | 2.01** | 0.13** |
| G x E | 1085 | 1060594.00** | 5.90** | 7.73** | 0.13ns | 346.13** | 93.71** | 0.78** | 0.81** | 0.56** | 1.18** | 0.9** | 0.08** |
| Error | 1048 | 653919 | 3.86 | 4.99 | 0.13 | 191.86 | 61.78 | 0.52 | 0.66 | 0.32 | 0.57 | 0.61 | 0.05 |

*, **, Significant at 0.05 and 0.01probability levels, respectively, and ns, non-significant.
