# Supplementary material for: Combining Abilities and Heterotic Patterns among Early Maturing Maize Inbred Lines under Optimal and Striga-Infested Environments
Source: Genes (Basel). 2022 Dec 5;13(12):2289. doi: 10.3390/genes13122289 (PMC9778638; doi:10.3390/genes13122289)
Supplement: Supplementary file 1 [file genes-13-02289-s001.zip › Supplementary Table 4.docx]

Supplementary Table 4. General combining ability effects of 30 inbred lines for grain yield, *Striga* damage rating and emerged *Striga* counts evaluated under contrasting environments, 2016–2017.

|  | *Striga* | | Optimal | | Across | | Striga damage (8WAP) | | Striga damage (10 WAP) | | Striga count (8 WAP) | | Striga count (10 WAP) | |
| --- | --- | --- | --- | --- | --- | --- | --- | --- | --- | --- | --- | --- | --- | --- |
| Inbred lines | YGCAf | YGCAm | YGCAF | YGCAm | YGCAf | YGCAm | GCAf | GCAm | GCAf | GCAm | GCAf | GCAm | GCAf | GCAm |
| TZEI 3A | -17.65 | -174.60 | -232.57 | 1.57 | -172.45 | -86.51 | 0.14 | 0.18 | 0.24 | 0.10 | 0.13 | 0.16 | 0.22 | 0.08 |
| TZEI 8 | 46.82 | 164.98 | 1.96 | 446.32** | 20.92 | 311.71** | -0.16 | -0.12 | 0.00 | 0.10 | 0.04 | 0.30* | 0.06 | 0.20 |
| TZdEI 40 | 175.23 | 219.43* | 790.67** | 909.59** | 507.27** | 602.88** | -0.12 | -0.08 | -0.05 | -0.12 | 0.23 | 0.14 | 0.26 | 0.15 |
| TZdEI 69 | -103.88 | -3.39 | 330.56 | 27.4 | 113.34 | 1.97 | -0.02 | 0.05 | 0.08 | -0.24 | 0.41* | 0.13 | 0.24 | 0.28 |
| TZdEI 124 | 296.23* | 128.49 | 416.11* | 316.51 | 356.17** | 222.5* | 0.02 | -0.14 | 0.24 | -0.07 | -0.42* | 0.13 | -0.46* | 0.18 |
| TZEI 127 | 286.94* | 219.29* | 436.73* | 583.5** | 379.19** | 397.93** | -0.36 | -0.07 | 0.17 | -0.39* | 0.02 | -0.62* | -0.04 | -0.61* |
| TZdEI 192 | -21.73 | 92.93 | 430.83* | -232.55 | 204.55 | -69.81 | -0.08 | 0.24 | -0.34* | 0.09 | -0.22 | -0.23 | -0.38* | -0.30* |
| TZdEI 215 | 233.35 | 114.08 | 596.29** | 250.94 | 404.79** | 182.51 | -0.31 | 0.09 | -0.10 | 0.02 | 0.02 | 0.38* | 0.06 | 0.28 |
| TZdEI 216 | 129.74 | 203.86* | -45.26 | 177.4 | 42.24 | 180.6 | -0.17 | -0.57** | -0.40* | -0.36* | 0.20 | -0.34* | 0.13 | -0.12 |
| TZdEI 238 | 80.88 | -34.38 | -218.34 | -13.7 | -68.73 | -24.04 | -0.16 | -0.11 | -0.07 | -0.18 | 0.16 | 0.04 | 0.15 | 0.03 |
| TZdEI 272 | -100.61 | 25.61 | 372.18* | 92.16 | 213.21 | 58.89 | -0.09 | -0.08 | -0.23 | -0.17 | 0.13 | -0.48* | -0.25 | -0.57* |
| TZdEI 283 | 46.38 | -73.02 | 142.14 | -230.58 | 94.26 | -161.83 | 0.22 | -0.25 | 0.23 | 0.00 | -0.17 | 0.32* | -0.14 | 0.18 |
| TZEI 298 | -186.45 | -102.98 | -360.29 | -214.02 | -283.4** | -158.5 | 0.39* | 0.20 | 0.15 | 0.10 | -0.09 | -0.31* | 0.02 | -0.16 |
| TZEI 365 | -136.73 | -66.06 | -338.52 | -312.9 | -237.63* | -189.47* | -0.05 | 0.11 | -0.02 | 0.27 | -0.21 | 0.04 | -0.12 | -0.06 |
| TZEI 376 | -218.66 | -121.00 | -290.08 | 242.64 | -254.37* | 60.82 | 0.27 | -0.10 | 0.19 | -0.12 | -0.15 | 0.02 | -0.10 | 0.14 |
| TZEI 378 | -183.79 | 30.10 | -274.62 | 197.27 | -229.21* | 103.66 | 0.03 | 0.08 | 0.37* | 0.01 | -0.05 | -0.32* | 0.14 | -0.41* |
| TZEI 379 | 111.53 | -157.55 | -152.82 | -171.49 | -20.64 | -124.4 | -0.06 | 0.69** | -0.29 | 0.59* | -0.38* | 0.22 | -0.37* | 0.08 |
| TZEI 402 | 71.35 | 137.87 | -375.6* | -130.65 | -162.15 | 3.61 | -0.13 | -0.39** | -0.06 | -0.05 | -0.20 | 0.26 | -0.04 | 0.36* |
| TZEI 449 | 76.22 | -148.38 | 456.77* | -262.45 | 293.9** | -229.73* | 0.10 | -0.34 | 0.03 | -0.12 | 0.05 | 0.06 | 0.23 | -0.05 |
| TZEI 456 | -100.00 | -125.79 | -12.46 | -83.86 | -16.84 | -54.38 | 0.18 | 0.02 | 0.28 | -0.05 | -0.13 | 0.04 | 0.09 | -0.14 |
| TZEI 461 | 145.07 | 42.95 | -242.62 | -450.91** | -132.93 | -213.45* | 0.17 | -0.10 | 0.19 | 0.11 | 0.52* | -0.37* | 0.44* | -0.09 |
| TZEI 462 | 58.56 | -210.8* | -97.31 | -245.63 | -25.6 | -231.68* | -0.14 | 0.40** | -0.19 | 0.26 | 0.00 | -0.43* | 0.18 | -0.30* |
| TZEI 467 | -154.03 | 65.18 | -343.68 | -142.79 | -295.42** | -42.27 | 0.14 | -0.14 | 0.12 | -0.11 | 0.02 | 0.18 | -0.08 | 0.11 |
| TZEI 468 | 41.28 | 46.30 | -72.26 | -145.07 | 40.58 | -85.08 | -0.12 | 0.01 | -0.07 | 0.08 | -0.49* | -0.03 | -0.39* | -0.01 |
| TZEI 470 | -43.84 | 203.35* | -335.76 | -186.17 | -229.86* | 15.65 | -0.10 | -0.09 | -0.25 | -0.37* | 0.03 | -0.49* | -0.10 | -0.40* |
| TZEI 472 | -91.49 | 52.13 | 16.72 | -111.22 | -41.32 | -69.6 | 0.17 | -0.22 | 0.18 | -0.27 | 0.10 | -0.41* | -0.15 | -0.27 |
| TZEI 475 | -38.41 | -88.04 | -34.18 | -212.82 | -16.71 | -196.04* | -0.20 | 0.37** | -0.01 | 0.16 | -0.13 | 0.34* | -0.08 | 0.26 |
| TZEI 485 | -121.83 | -157.28 | -139.47 | -20.47 | -120.69 | -50.5 | -0.16 | 0.00 | -0.32 | -0.05 | -0.22 | -0.03 | -0.20 | -0.21 |
| TZEI 497 | -40.77 | -131.93 | -88.79 | 215.13 | -68.24 | 98.41 | 0.37 | 0.18 | 0.22 | 0.23 | 0.01 | -0.21 | -0.11 | -0.01 |
| TZEI 520 | -239.77 | -151.41 | -336.31 | -293.13 | -294.23** | -253.84** | 0.23 | 0.17 | 0.25 | -0.02 | -0.03 | 0.28 | -0.11 | 0.15 |
| SE | 110.28 | 86.74 | 184.59 | 164.30 | 109.16 | 95.37 | 0.17 | 0.16 | 0.16 | 0.15 | 0.17 | 0.14 | 0.12 | 0.13 |

^*^, ^**^, Significant at 0.05 and 0.01probability levels, respectively, YGCAm and YGCAf, general combining ability (female and male) for grain yield; GCAm and GCAf, general combining ability for male and female, respectively.
